# Mid-Term Oncological Outcomes of Vaginal Natural Orifice Transluminal Endoscopic Surgery Compared with Total Laparoscopic Hysterectomy for Early-Stage Endometrial Cancer: A Single-Center Retrospective Study

**DOI:** 10.3390/jcm15093350

**Published:** 2026-04-28

**Authors:** Ken Imai, Junya Abe, Kenro Chikazawa, Mina Hasegawa, Nanami Suzuki, Miyuki Taniguchi, Tomoyuki Kuwata

**Affiliations:** Department of Obstetrics and Gynecology, Saitama Medical Center, Jichi Medical University, 1-847 Amanuma-cho, Omiya-ku, Saitama 330-8503, Japan; kunkun42@jichi.ac.jp (K.I.); r2202aj@jichi.ac.jp (J.A.); m1600082@gunma-u.ac.jp (M.H.); nssb.9761@gmail.com (N.S.); mx.oner.abc@icloud.com (M.T.); obstet3w@gmail.com (T.K.)

**Keywords:** endometrial cancer, vNOTES, total laparoscopic hysterectomy, oncological safety, oncological outcomes

## Abstract

**Background/Objectives**: Vaginal natural orifice transluminal endoscopic surgery (vNOTES) is increasingly being used to avoid abdominal incisions; however, its mid-term oncological safety in endometrial cancer remains unclear. **Methods**: This single-center retrospective cohort study included patients with International Federation of Gynecology and Obstetrics (FIGO) clinical stage IA endometrioid endometrial carcinoma undergoing simple hysterectomy between January 2014 and December 2023. Patients were treated with either total laparoscopic hysterectomy (TLH) or vNOTES. Patients who underwent lymph node assessment were excluded. Follow-up assessed mid-term oncological outcomes. Recurrence-free survival (RFS) was evaluated using the Kaplan–Meier method and compared between the groups, and Cox proportional hazards models were used to identify prognostic factors for RFS. **Results**: In total, 130 patients were included: 109 underwent TLH and 21 vNOTES. The median follow-up period was 48 and 33 months in the TLH and vNOTES groups, respectively. Postoperative adjuvant therapy was more frequent in the vNOTES group. The operative time was significantly shorter with vNOTES. Postoperative complications were low and similar between the groups. The 3-year RFS was 92.8% and 94.4% in the TLH and vNOTES groups, respectively, without a significant difference (*p* = 0.874). Lymphovascular space invasion was significantly associated with worse RFS, whereas surgical approach was not significantly associated with RFS. **Conclusions**: No statistically significant difference in mid-term RFS was observed between vNOTES hysterectomy and conventional TLH in this highly selected low-risk cohort. However, the study was underpowered and subject to residual confounding; therefore, these findings should be considered preliminary and hypothesis-generating.

## 1. Introduction

Endometrial cancer is the most common gynecological malignancy in developed countries and the second most common in developing countries. In 2020, approximately 417,367 new cases of endometrial cancer were diagnosed worldwide, with 97,370 reported deaths and a 5-year prevalence rate of 33.9% (1,415,213 cases) [1].

Large randomized controlled trials and other studies from multiple countries have demonstrated that laparoscopic surgery provides oncological outcomes comparable to those of open surgery in patients with early-stage endometrial cancer [2,3]. Based on this evidence, the European Society of Gynaecological Oncology (ESGO), European Society for Radiotherapy and Oncology (ESTRO), and European Society of Pathology (ESP) in their 2020 guidelines, jointly recommended minimally invasive surgery as the preferred surgical approach for early-stage endometrial cancer, including selected high-risk patients [4].

However, patients with endometrial cancer frequently present with obesity and advanced age, and abdominal wall-related complications remain a clinical concern, even with minimally invasive surgery. These include wound infection, wound dehiscence, and incisional hernia, as well as port-related complications such as bleeding, port-site hernia, and postoperative pain. To reduce these procedure-related morbidities, increasing attention has been directed toward surgical approaches that avoid abdominal wall incisions.

Minimally invasive gynecologic surgery has evolved from conventional laparoscopy to single-port laparoscopy and, more recently, to vaginal natural orifice transluminal endoscopic surgery (vNOTES) [5]. Because vNOTES does not require abdominal wall incisions, it may offer potential advantages in patients with obesity by minimizing abdominal wall- and port-related complications, in addition to contributing to reduced postoperative pain and faster recovery.

Recently, several studies have reported the feasibility and perioperative safety of vNOTES-based staging surgery for early-stage endometrial cancer [6,7,8,9,10]. Nevertheless, most of these studies focused on short-term outcomes, and data on mid- to long-term oncological outcomes remain limited. In particular, comparative studies evaluating the oncological outcomes of conventional total laparoscopic hysterectomy (TLH) and vNOTES are scarce.

Additionally, although minimally invasive approaches have been widely accepted for early-stage endometrial cancer, the introduction of novel surgical techniques requires careful validation of oncological safety [2,3]. In particular, given the concerns raised in other gynecologic malignancies regarding potential tumor dissemination associated with minimally invasive surgery [11], it remains essential to confirm that new approaches such as vNOTES do not adversely affect oncological outcomes. However, evidence regarding mid-term oncological outcomes following vNOTES hysterectomy remains scarce, especially in comparison with established laparoscopic techniques.

Therefore, in this study, we aimed to evaluate the mid-term oncological safety of the vNOTES approach for early-stage endometrial cancer in comparison with TLH.

## 2. Materials and Methods

### 2.1. Study Design and Ethical Approval

This study was conducted in accordance with the principles outlined in the Declaration of Helsinki and was approved by the Institutional Review Board (IRB) of our institution (IRB approval number: S25-142). The requirement for written informed consent was waived owing to the retrospective design of the study. This study complies with the Strengthening the Reporting of Observational Studies in Epidemiology (STROBE) guidelines. All data were retrieved from the institutional cancer registry and are available for validation or comparative research upon reasonable request.

### 2.2. Patients and Eligibility Criteria

This single-center retrospective study included women who underwent primary surgical treatment with simple hysterectomy for International Federation of Gynecology and Obstetrics (FIGO) clinical stage IA endometrioid endometrial carcinoma between January 2014 and December 2023. Follow-up data were censored on 31 December 2025 to ensure a minimum potential postoperative follow-up of 24 months for all included patients. This design was intended to ensure adequate assessment of mid-term oncological outcomes.

In Japan, the national health insurance coverage for minimally invasive surgery for endometrial cancer was limited to preoperative stage IA disease during the study period. vNOTES was introduced at our institution in 2020 and was subsequently performed in parallel with conventional TLH. Consequently, although the two surgical approaches were not evenly distributed throughout the study period, patients in both groups were treated during partially overlapping time periods. The surgical approach was selected at the discretion of the treating surgeons based on institutional practice and preoperative clinical assessment. In general, vNOTES was considered for patients in whom transvaginal access appeared feasible, including those with adequate vaginal exposure and without obvious evidence of pelvic adhesions on pelvic examination and ultrasonography. Surgeons experienced in vaginal surgery were responsible for the introduction and performance of vNOTES.

Patients were excluded if they met any of the following criteria: a history of other malignancies; prior treatment with definitive radiotherapy, chemoradiotherapy, or neoadjuvant chemotherapy; or lymph node assessment, which represented a different surgical procedure and risk population.

### 2.3. Surgical Procedures

In the conventional TLH procedure, a camera trocar was inserted through the umbilicus, followed by coagulation and transection of the fallopian tubes and collection of peritoneal washings. Subsequent surgical steps included transection of the round ligament, identification of the ureter with division of the infundibulopelvic ligament, transection of the uterosacral ligaments, bladder dissection, colpotomy, and closure of the vaginal cuff, as previously described [12]. The use of a uterine manipulator was at the surgeon’s discretion. When used, it was inserted only after tubal coagulation. To minimize the risk of cancer cell spillage, Z-suture closure of the cervix, vaginal irrigation before and after colpotomy, and specimen retrieval in a protective bag were routinely performed [13].

In the vNOTES procedure, one ampule of vasopressin (20 units) and 20 mg of indigo carmine were diluted in 100 mL of normal saline prior to hydrodissection. For vaginal colpotomy, 7–10 mL of the diluted vasopressin solution was injected at 3–4 points around the cervix, while avoiding the myometrium [14]. Posterior and anterior colpotomy were then performed, and peritoneal washings were collected. A Z-suture was placed on the cervix before proceeding further. A GelPOINT^®^ V-Path platform (Applied Medical, Rancho Santa Margarita, CA, USA) with three trocars was inserted transvaginally. The ureters were identified, followed by transection of the parametrium, round ligaments, and infundibulopelvic ligaments. The surgical specimen was removed transvaginally, and the vaginal cuff was closed.

### 2.4. Adjuvant Therapy and Follow-Up

Postoperative adjuvant therapy was administered according to the guidelines of the Japan Society of Gynecologic Oncology [15]. After treatment completion, the patients were followed up at regular intervals to monitor for recurrence and complications. Vaginal stump cytology and transvaginal ultrasonography were performed every 3 months for the first 2 postoperative years, and every 6 months thereafter for an additional 3 years. Computed tomography was performed every 6 months.

### 2.5. Statistical Analysis

Statistical analyses were performed using IBM SPSS Statistics for Windows (version 26.0; IBM Corp., Armonk, NY, USA). Continuous variables were compared using Student’s *t*-test for normally distributed data and the Wilcoxon rank-sum test for non-normally distributed data. Categorical variables were analyzed using Fisher’s exact test.

Recurrence-free survival (RFS) was estimated using the Kaplan–Meier method and compared between the two groups using the log-rank test. A Cox proportional hazards model including pathological upstaging, tumor histology, lymphovascular space invasion, and surgical approach was used to identify potential prognostic factors. Hazard ratios (HRs) were reported with 95% confidence intervals. A two-sided *p*-value <0.05 was considered statistically significant. Variables for the Cox model were selected based on clinical relevance and prior literature [3]. Ascites cytology was excluded because no recurrence events occurred among patients with negative cytology, precluding reliable estimation. Given the limited number of recurrence events, the number of covariates was restricted to avoid overfitting. Postoperative adjuvant therapy was not included in the primary model because it was determined after surgery according to pathological risk factors; instead, an additional model including adjuvant therapy was performed as a sensitivity analysis [4,16,17].

## 3. Results

During the study period, 248 patients with FIGO stage IA endometrial cancer underwent minimally invasive surgery. Among them, 94 patients who underwent lymph node assessment, 22 treated with robot-assisted hysterectomy, and two with concomitant malignancies were excluded. Consequently, 130 patients were included in the final analysis, comprising 109 patients in the TLH group and 21 patients in the vNOTES group.

The median follow-up duration was 48 and 33 months in the TLH and vNOTES groups, respectively.

Patient characteristics are summarized in Table 1. The operative time was significantly shorter in the vNOTES group. Baseline demographic characteristics were broadly similar between the two groups; however, postoperative adjuvant therapy was more frequent in the vNOTES group, and pathological upstaging was numerically more frequent in the vNOTES group, although the difference did not reach statistical significance.

Postoperative complications did not differ significantly between the groups (Table 2). In the TLH group, urinary tract infection occurred in one patient (0.9%), and surgical site infection occurred in two patients (1.8%). In the vNOTES group, one patient (4.8%) developed an intra-abdominal abscess. No severe complications, including organ injuries, were observed in either group.

In the overall cohort, recurrence occurred in eight patients in the TLH group and one patient in the vNOTES group. In the TLH group, recurrence sites included the vaginal cuff in two patients, multiple peritoneal dissemination in one, liver metastasis in one, bone metastasis in one, lung metastasis in one, multiple metastases involving the pelvic and para-aortic lymph nodes and liver in one, and multiple metastases involving the pelvic and para-aortic lymph nodes and spleen in one. The single recurrence in the vNOTES group was a vaginal cuff recurrence. The time to recurrence for each case is summarized in Supplementary Table S1.

**Figure 1:**
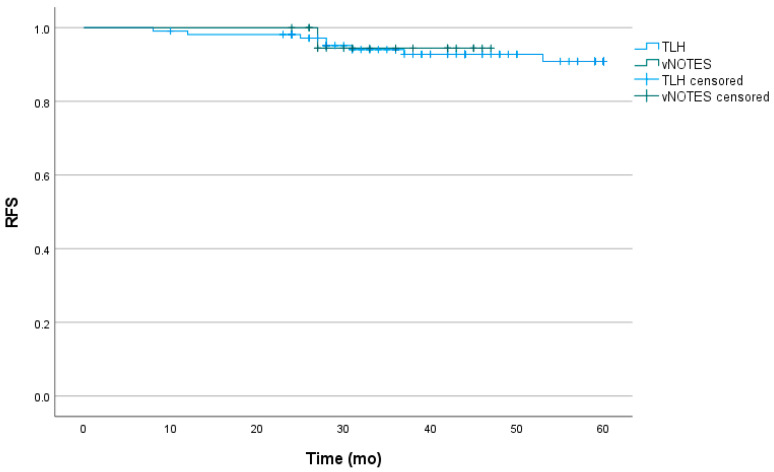
Recurrence-free survival (RFS). The 3-year RFS rates were 92.8% and 94.4% in the TLH and vNOTES groups, respectively, with no significant difference between the groups (*p* = 0.874). TLH, total laparoscopic hysterectomy; vNOTES, vaginal natural orifice transluminal endoscopic surgery.

The 3-year RFS rates were 92.8% and 94.4% in the TLH and vNOTES groups, respectively, with no significant difference between the groups (*p* = 0.874; Figure 1).

On multivariable Cox proportional hazards regression analysis, lymphovascular space invasion was the only significant prognostic factor for RFS, whereas pathological upstaging showed a nonsignificant trend (Table 3). Ascites cytology was not included in the Cox proportional hazards model because no recurrence events occurred among patients with negative cytology, resulting in complete separation and an unreliable estimation.

Because postoperative adjuvant therapy differed between the groups, we additionally performed a multivariable Cox analysis including adjuvant therapy. In this model, surgical approach remained unassociated with RFS (Supplementary Table S2). Moreover, lymphovascular space invasion no longer reached statistical significance, although a trend toward worse RFS remained (HR: 4.704; 95% CI: 0.879–25.174; *p* = 0.070).

To reduce potential temporal bias related to the later introduction of vNOTES, we performed a sensitivity analysis restricted to patients treated from 2020 onward. This analysis included 56 and 21 patients treated with TLH and vNOTES, respectively. The median follow-up was 42 months in the TLH group and 33 months in the vNOTES group. Recurrence occurred in three patients in the TLH group and one patient in the vNOTES group. In the TLH group, recurrence sites included the vaginal cuff, lung, and multiple metastases involving the pelvic lymph nodes, para-aortic lymph nodes, and liver, whereas the single recurrence in the vNOTES group occurred at the vaginal cuff. The Kaplan–Meier analysis showed no significant difference in RFS between the groups (Supplementary Figure S1, log-rank *p* = 0.956). In supplementary Cox regression analyses, surgical approach was not significantly associated with RFS either in the base multivariable model or in the model additionally including adjuvant therapy (Supplementary Tables S3 and S4).

## 4. Discussion

In the present study, the vNOTES approach for hysterectomy demonstrated no significant difference in the mid-term RFS compared with conventional TLH. Cox regression analysis identified lymphovascular space invasion as a significant prognostic factor for RFS, whereas pathological upstaging showed a nonsignificant trend. In contrast, the surgical approach itself was not a significant determinant of oncological outcomes; that is, in patients with FIGO stage IA endometrial cancer, the prognosis in this highly selected cohort appeared to be more strongly influenced by tumor-related factors than by the route of hysterectomy.

In endometrial cancer, multiple large randomized controlled trials have demonstrated the oncological safety of minimally invasive surgery in early-stage disease, positioning it differently from cervical cancer, wherein concerns regarding minimally invasive approaches have been raised [2,11]. Nevertheless, concerns regarding intraperitoneal tumor dissemination have not been completely eliminated, and cautious surgical management is particularly important when introducing novel surgical techniques [18]. In this study, tumor spillage prevention strategies were consistently implemented, including early tubal coagulation, Z-suture closure of the cervix, vaginal irrigation before and after colpotomy, and minimization of direct specimen manipulation. These measures may have contributed, at least in part, to the preservation of oncological outcomes in the vNOTES group.

Although studies on vNOTES for endometrial cancer have increased in recent years, most have focused primarily on perioperative outcomes such as operative time, blood loss, and length of hospital stay, and data addressing oncological outcomes remain limited [6,7,8,9,10]. Although the follow-up duration in the present study was insufficient for drawing definitive long-term conclusions, the data on mid-term RFS complement previous feasibility-focused reports.

Notably, although pathological upstaging tended to be more common in the vNOTES group, no deterioration in RFS was observed. This finding may suggest that postoperative adjuvant therapy was administered according to established oncological risk factors and that the surgical approach did not preclude appropriate postoperative management [2,4,19]. In this context, the vNOTES approach may be feasible in selected patients.

FIGO stage IA endometrial cancer could be considered potentially suitable for the vNOTES approach, as lymph node assessment can often be omitted and the uterine size is typically small [2]. For surgeons experienced in vaginal surgery, vNOTES may represent a feasible minimally invasive option; however, careful training and stepwise implementation remain essential. Furthermore, early-stage endometrial cancer is associated with an inherently favorable prognosis, and satisfactory oncological outcomes can often be achieved regardless of the hysterectomy approach, supporting the findings of this study.

The present findings suggest that vNOTES may represent a feasible minimally invasive surgical option for selected patients with early-stage endometrial cancer; however, its safe implementation requires appropriate patient selection, standardization of surgical techniques including tumor spillage–prevention measures, and adequate surgeon training. In vNOTES, posterior entry into the pouch of Douglas may be relatively straightforward because recognizable anatomical landmarks, such as Denonvilliers’ fascia can be identified [20]. In contrast, anterior dissection of the vesicouterine pouch is more complex, as multiple dissectible layers exist, and incorrect plane selection may occur, necessitating careful layer identification. Indeed, recent anatomical studies have demonstrated that no single, well-defined septal structure exists in the vesicouterine space, confirming that the choice of dissection plane directly affects surgical safety [21]. Therefore, for the wider adoption of vNOTES, stepwise implementation by surgeons experienced in vaginal surgery, together with simulation-based training and structured educational programs grounded in detailed anatomical understanding, will be essential to ensure oncological safety [22].

Although lymph node assessment using vNOTES was not performed in this study, reports describing vNOTES-based lymphadenectomy and sentinel lymph node biopsy have recently emerged [23,24,25]. Further studies with long-term follow-up are warranted to evaluate the oncological safety of these procedures.

This study has several limitations. First, the sample size was limited, particularly in the vNOTES group, and follow-up was also shorter in the vNOTES group. Post hoc power calculations based on assumed 5% and 10% absolute differences in 3-year RFS yielded low power estimates (0.120 and 0.332, respectively), indicating that the study was underpowered to detect clinically meaningful differences. Therefore, the absence of a statistically significant difference should not be interpreted as evidence of equivalence. Second, selection bias cannot be excluded as this was a retrospective single-center study and vNOTES was preferentially performed in patients considered suitable for transvaginal access, such as those with adequate vaginal exposure and no obvious preoperative evidence of pelvic adhesions. Third, because vNOTES was introduced later in the study period, temporal bias related to surgical experience, perioperative management, and adjuvant treatment strategies may also have influenced the results. Fourth, 94 of the 248 initially screened patients (37.9%) who underwent lymph node assessment were excluded, resulting in a highly selected low-risk cohort and limiting the generalizability of the findings to the broader population of patients with clinical stage IA endometrial cancer. Finally, the cohort was restricted to patients treated till December 2023 to ensure at least 2 years of potential follow-up for all included patients.

## 5. Conclusions

In this retrospective single-center study of a highly selected low-risk cohort, no statistically significant difference in mid-term RFS was observed between vNOTES hysterectomy and conventional TLH for FIGO stage IA endometrial cancer. However, given the small sample size, shorter follow-up in the vNOTES group, exclusion of patients who underwent lymph node assessment, and potential residual confounding, these findings should be considered preliminary and hypothesis-generating.

## Figures and Tables

**Table 1:** Patient characteristics.

	TLH (*N* = 109)	vNOTES (*N* = 21)	*p*-Value
Age (years)	58.7 ± 13.4	61.1 ± 10.4	0.435
BMI	26.1 ± 6.1	25.0 ± 6.1	0.473
Preoperative tumor diameter (mm)	27.1 ± 25.0	34.3 ± 37.7	0.267
Postoperative stage			0.054
IA	93 (85.3%)	16 (76.2%)	
IB	15 (13.8%)	3 (14.3%)	
II	1 (0.9%)	2 (9.5%)	
Pathology			0.529
Endometrioid G1–2	100 (91.7%)	18 (85.7%)	
Endometrioid G3	2 (1.8%)	0	
Serous	2 (1.8%)	1 (4.8%)	
Clear cell	2 (1.8%)	0	
carcinosarcoma	1 (0.9%)	1 (4.8%)	
Mixed	2 (1.8%)	1 (4.8%)	
Operative time (min)	158.0 ± 54.9	89.2 ± 29.1	<0.001
Blood loss (g)	108.1 ± 161.7	46.9 ± 69.4	0.091
Lymphovascular invasion	13 (12.0%)	4 (19.0%)	0.478
Positive ascites cytology	12 (11.5%)	3 (20.0%)	0.402
Adjuvant therapy	22 (20.2%)	9 (42.9%)	0.046 *

BMI, body mass index; vNOTES, vaginal natural orifice transluminal endoscopic surgery; TLH, total laparoscopic hysterectomy. * *p* < 0.05.

**Table 2:** Surgery-related complications.

	TLH (*N* = 109)	vNOTES (*N* = 21)	*p*-Value
Urinary tract infection	1 (0.9%)	0	1
Intra-abdominal abscess	0	1 (4.8%)	0.162
Wound infection	2 (1.8%)	0	1
Organ injury	0	0	

vNOTES, vaginal natural orifice transluminal endoscopic surgery; TLH, total laparoscopic hysterectomy.

**Table 3:** Multivariable Cox regression analysis of recurrence-free survival.

	HR	95% Confidence Interval	*p*-Value
vNOTES ref TLH	0.768	0.268–2.199	0.623
Upstaged	3.905	0.787–19.384	0.096
Non endometrioid G1–2	1.040	0.185–5.834	0.965
Lymphovascular invasion	5.067	1.056–24.323	0.043 *

HR, hazard ratio; vNOTES, vaginal natural orifice transluminal endoscopic surgery; TLH, total laparoscopic hysterectomy. * *p* < 0.05.

## Data Availability

The data that support the findings of this study are available from the corresponding author upon reasonable request.

## Supplementary Materials

The following supporting information can be downloaded at: https://www.mdpi.com/article/10.3390/jcm15093350/s1, Table S1: Recurrence site and time (months); Table S2: Cox regression analysis of recurrence-free survival, additionally adjusted for postoperative adjuvant therapy; Table S3: Multivariable Cox regression analysis of recurrence-free survival in patients treated from 2020 onward; Table S4: Multivariable Cox regression analysis of recurrence-free survival in patients treated from 2020 onward, additionally adjusted for postoperative adjuvant therapy; Figure S1: Recurrence-free survival (RFS) of patients treated from 2020 onward. The 3-year RFS rates were 94.5% and 94.4% in the TLH and vNOTES groups, respectively, with no significant difference between the groups (*p* = 0.956). TLH, total laparoscopic hysterectomy; vNOTES, vaginal natural orifice transluminal endoscopic surgery.

## Abbreviations

The following abbreviations are used in this manuscript:

vNOTES	Vaginal natural orifice transluminal endoscopic surgery
TLH	Total laparoscopic hysterectomy
FIGO	International Federation of Gynecology and Obstetrics
RFS	Recurrence-free survival
HR	Hazard ratio
