# Supplementary material for: Mid-Term Oncological Outcomes of Vaginal Natural Orifice Transluminal Endoscopic Surgery Compared with Total Laparoscopic Hysterectomy for Early-Stage Endometrial Cancer: A Single-Center Retrospective Study
Source: J Clin Med. 2026 Apr 28;15(9):3350. doi: 10.3390/jcm15093350 (PMC13164299; doi:10.3390/jcm15093350)
Supplement: Supplementary file 1 [file jcm-15-03350-s001.zip › jcm-4247690-supplementary.pdf]

Supplementary Table S1. Recurrence site and time (months)

Group	Recurrence site	Time to recurrence (months)
TLH	Vaginal cuff	12
TLH	Vaginal cuff	25
TLH	Multiple peritoneal dissemination	31
TLH	Liver metastasis	37
TLH	Bone metastasis	28
TLH	Lung metastasis	28
TLH	PLN, PAN, liver	8
TLH	PLN, PAN, spleen	53
vNOTES	Vaginal cuff	27

vNOTES, vaginal natural orifice transluminal endoscopic surgery; TLH, total laparoscopic hysterectomy; PLN, pelvic lymph node; PAN para-aortic lymph node.

Supplementary Table S2. Cox regression analysis of recurrence-free survival, additionally adjusted for postoperative adjuvant therapy

	HR	95% confidence interval	*P*-value
vNOTES ref TLH	0.751	0.258–2.188	0.600
Upstaged	3.611	0.646–20.179	0.144
Non endometrioid G1-2	1.090	0.191–6.228	0.923
Lymphovascular invasion	4.704	0.879–25.174	0.070
Adjuvant treatment	1.227	0.199-7.556	0.826

HR, hazard ratio; vNOTES, vaginal natural orifice transluminal endoscopic surgery; TLH, total laparoscopic hysterectomy. *$P < 0.05$

Supplementary Table S3. Multivariable Cox regression analysis of recurrence-free survival in patients treated from 2020 onward

	HR	95% confidence interval	*P*-value
vNOTES ref TLH	0.873	0.279–2.726	0.815
Upstaged	2.809	0.331–23.821	0.344
Lymphovascular invasion	3.832	0.452–32.476	0.218

HR, hazard ratio; vNOTES, vaginal natural orifice transluminal endoscopic surgery; TLH, total laparoscopic hysterectomy. *$P < 0.05$

Supplementary Table S4. Multivariable Cox regression analysis of recurrence-free survival in patients treated from 2020 onward, additionally adjusted for postoperative adjuvant therapy

	HR	95% confidence interval	*P*-value
vNOTES ref TLH	0.829	0.259–2.655	0.600
Upstaged	2.273	0.212–24.345	0.497
Non endometrioid G1-2	1.792	0.136–23.705	0.658
Lymphovascular invasion	3.304	0.239–38.446	0.392
Adjuvant treatment	1.749	0.053-57.952	0.754

HR, hazard ratio; vNOTES, vaginal natural orifice transluminal endoscopic surgery; TLH, total laparoscopic hysterectomy. $^{*}P < 0.05$

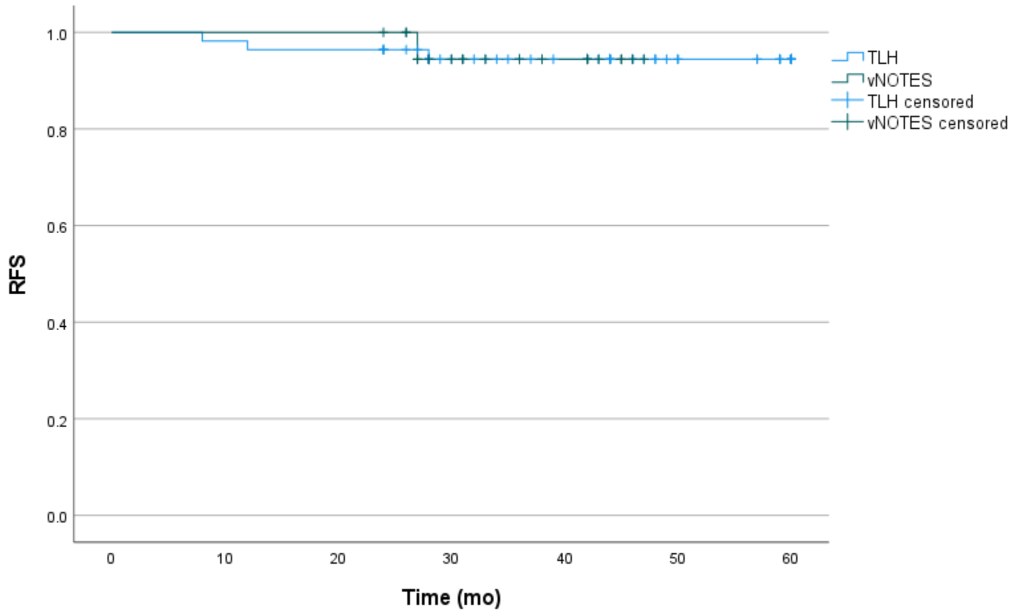

Supplementary Figure S1. Recurrence-free survival (RFS) of patients treated from 2020 onward. The 3-year RFS rates were 94.5% and 94.4% in the TLH and vNOTES groups, respectively, with no significant difference between the groups (P = 0.956). TLH, total laparoscopic hysterectomy; vNOTES, vaginal natural orifice transluminal endoscopic surgery.
